# Supplementary material for: Immunity-and-matrix-regulatory cells derived from human embryonic stem cells safely and effectively treat mouse lung injury and fibrosis
Source: Cell Res. 2020 Jun 16;30(9):794–809. doi: 10.1038/s41422-020-0354-1 (PMC7296193; doi:10.1038/s41422-020-0354-1)
Supplement: Supplementary file 1 — Supplementary Figure S1 [file 41422_2020_354_MOESM1_ESM.pdf]

Figure S1

a

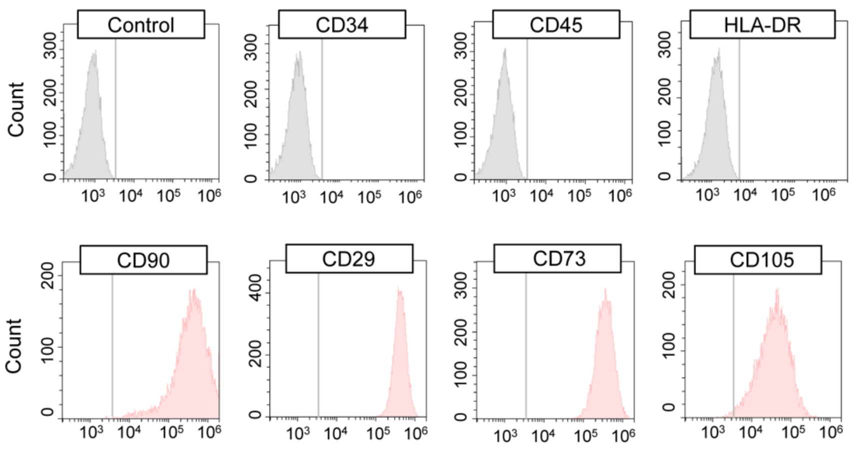

b

	CD73	CD90	CD29	CD105	CD34	CD45	HLA-DR
IMRCs	99.98±0.01	99.79±0.09	99.93±0.02	99.65±0.12	0.26±0.06	0.28±0.04	0.05±0.01
UCMSCs	99.76±0.12	96.46±0.32	99.97±0.01	97.73±0.06	0.03±0.01	0.44±0.15	0.02±0.01

c

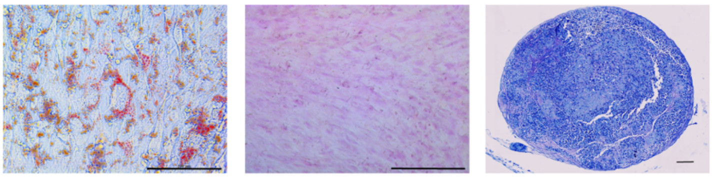

Fig. S1 Derivation of Immunity- and Matrix-Regulatory Cells (IMRCs) from human embryonic stem cells (hESCs).

a UCMSCs' expression of MSC-specific surface markers was determined by flow cytometry. Isotype control antibodies were used as controls for gating. The UCMSCs are $CD34^-$/$CD45^-$/HLA-DR^-/$CD90^+$/$CD29^+$/$CD73^+$/$CD105^+$ cells. **b** Quantification of flow cytometry for IMRCs and UCMSCs. **c** Representative staining of IMRCs after they were induced to undergo adipogenic differentiation (Oil Red O), osteogenic differentiation (Alizarin Red), and chondrogenic differentiation (Alcian Blue). Scale bars, 100 μm.
