# Supplementary material for: Immunity-and-matrix-regulatory cells derived from human embryonic stem cells safely and effectively treat mouse lung injury and fibrosis
Source: Cell Res. 2020 Jun 16;30(9):794–809. doi: 10.1038/s41422-020-0354-1 (PMC7296193; doi:10.1038/s41422-020-0354-1)
Supplement: Supplementary file 2 — Supplementary Figure S2 [file 41422_2020_354_MOESM2_ESM.pdf]

Figure S2

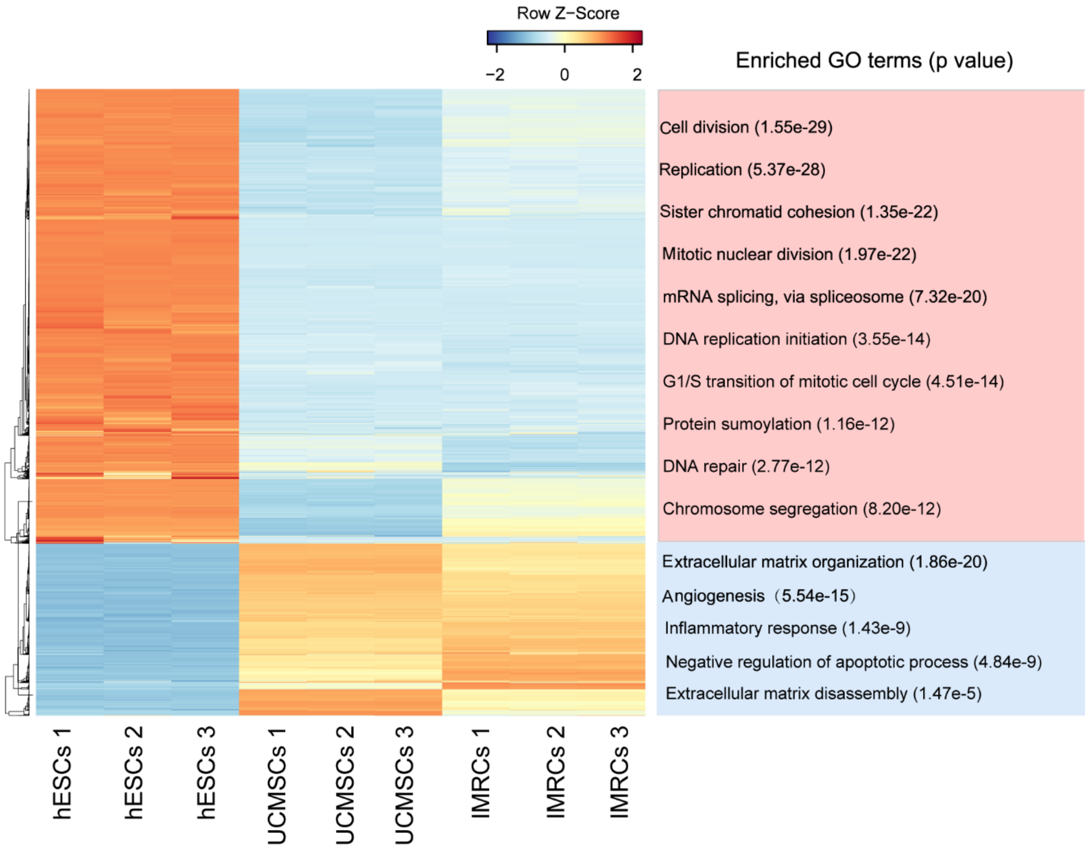

Fig. S2 The differentially expressed genes among hESCs and UCMSCs, IMRCs. In total, 4,730 differentially expressed genes were found in hESCs compared to UCMSCs and IMRCs. The enriched Gene Ontology (GO) terms and corresponding p values are shown.
