# Supplementary material for: Immunity-and-matrix-regulatory cells derived from human embryonic stem cells safely and effectively treat mouse lung injury and fibrosis
Source: Cell Res. 2020 Jun 16;30(9):794–809. doi: 10.1038/s41422-020-0354-1 (PMC7296193; doi:10.1038/s41422-020-0354-1)
Supplement: Supplementary file 3 — Supplementary Figure S3 [file 41422_2020_354_MOESM3_ESM.pdf]

Figure S3

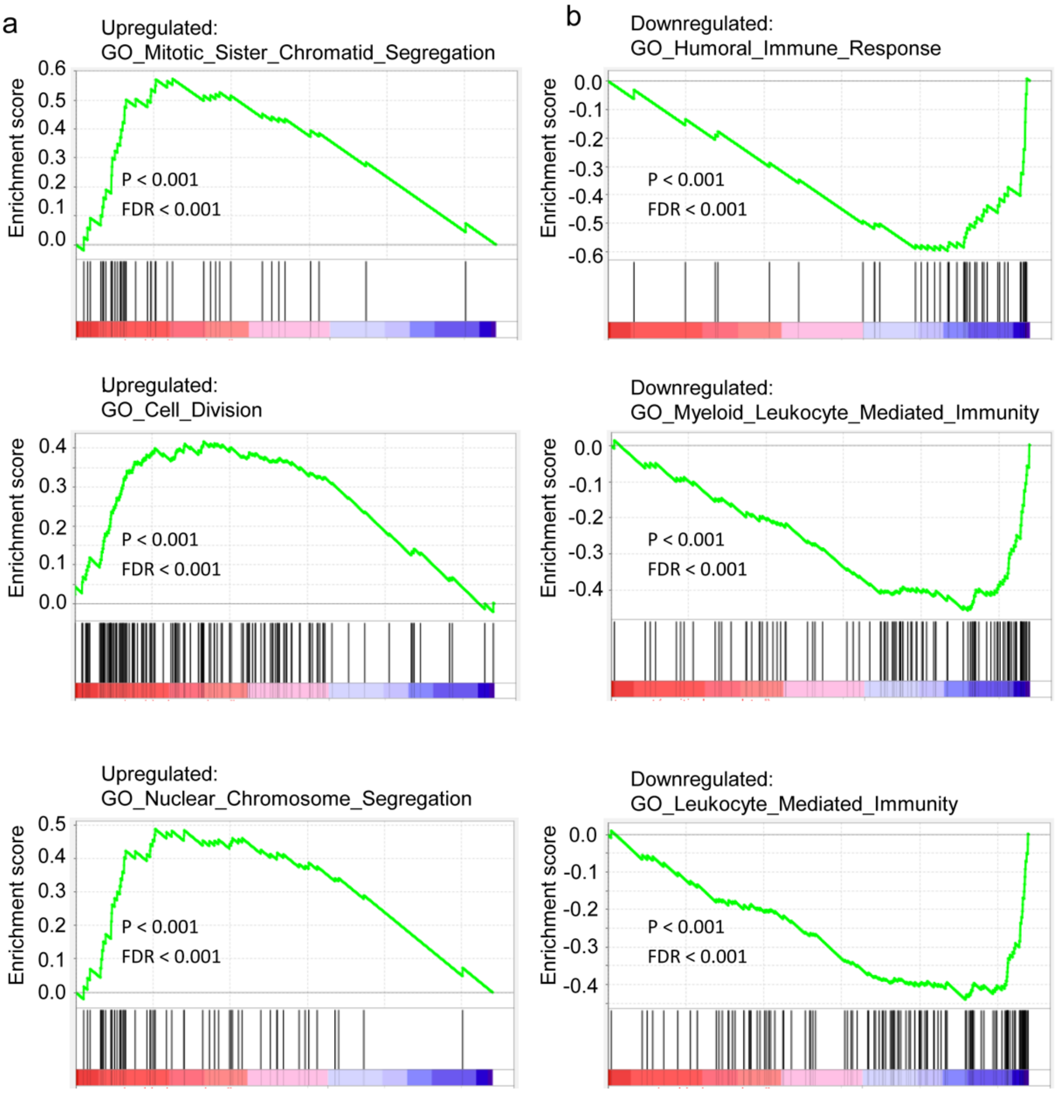

Fig. S3 IMRCs possess unique gene expression characteristics.

a Gene set enrichment analysis (GSEA) of the top up-regulated gene signature in IMRCs, compared with primary UCMSCs. **b** Gene set enrichment analysis (GSEA) of the top down-regulated gene signature in IMRCs, compared with primary UCMSCs.
