# Supplementary material for: Immunity-and-matrix-regulatory cells derived from human embryonic stem cells safely and effectively treat mouse lung injury and fibrosis
Source: Cell Res. 2020 Jun 16;30(9):794–809. doi: 10.1038/s41422-020-0354-1 (PMC7296193; doi:10.1038/s41422-020-0354-1)
Supplement: Supplementary file 4 — Supplementary Figure S4 [file 41422_2020_354_MOESM4_ESM.pdf]

Figure S4

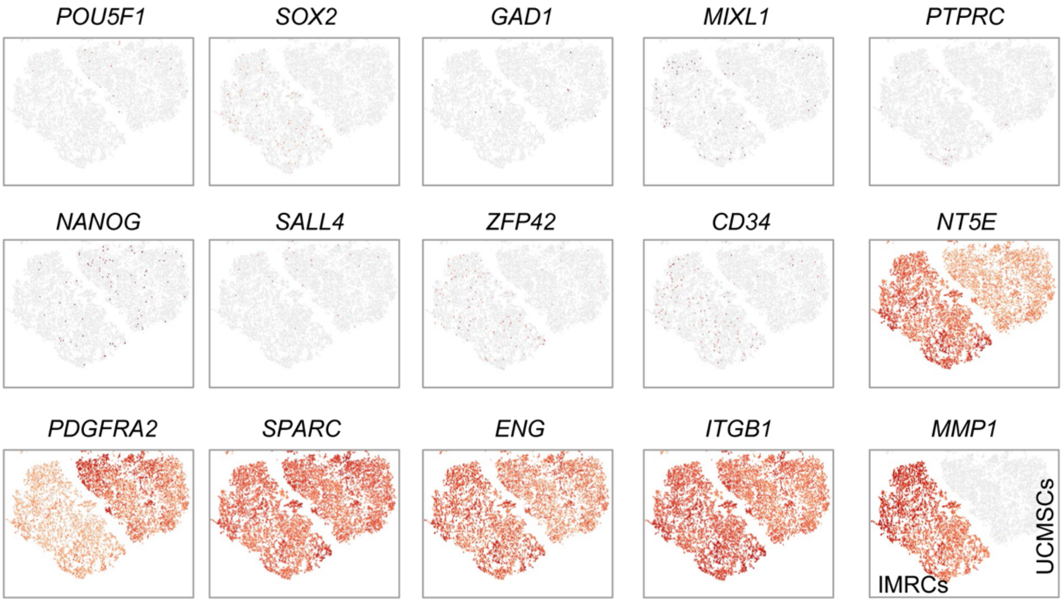

Fig. S4 IMRCs possess unique gene expression characteristics.

Heatmaps of specific gene expression amongst IMRCs and UCMSCs as measured by single cell RNAseq.
