# Supplementary material for: Immunity-and-matrix-regulatory cells derived from human embryonic stem cells safely and effectively treat mouse lung injury and fibrosis
Source: Cell Res. 2020 Jun 16;30(9):794–809. doi: 10.1038/s41422-020-0354-1 (PMC7296193; doi:10.1038/s41422-020-0354-1)
Supplement: Supplementary file 5 — Supplementary Figure S5 [file 41422_2020_354_MOESM5_ESM.pdf]

Figure S5

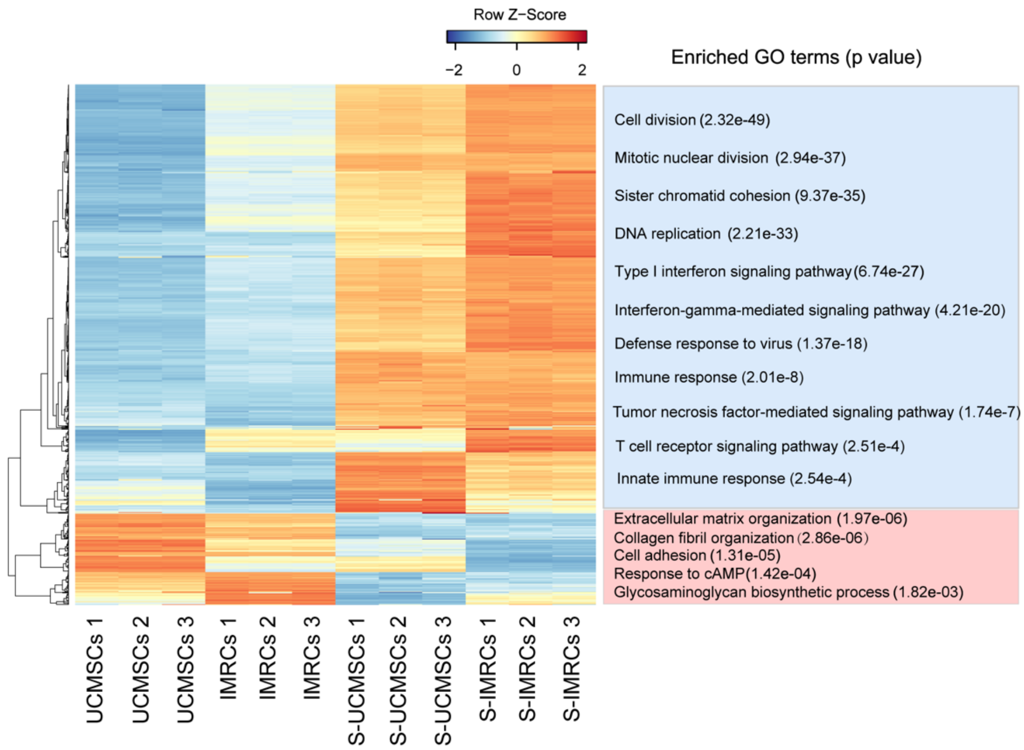

Fig. S5 The differentially expressed genes in UCMSCs and IMRCs, before and after IFN-γ stimulation. In total, 763 differentially expressed genes were found in UCMSCs and IMRCs before and after IFN-γ stimulation. The enriched Gene Ontology (GO) terms and corresponding p values are shown.
