# Supplementary material for: Immunity-and-matrix-regulatory cells derived from human embryonic stem cells safely and effectively treat mouse lung injury and fibrosis
Source: Cell Res. 2020 Jun 16;30(9):794–809. doi: 10.1038/s41422-020-0354-1 (PMC7296193; doi:10.1038/s41422-020-0354-1)
Supplement: Supplementary file 6 — Supplementary Figure S6 [file 41422_2020_354_MOESM6_ESM.pdf]

Figure S6

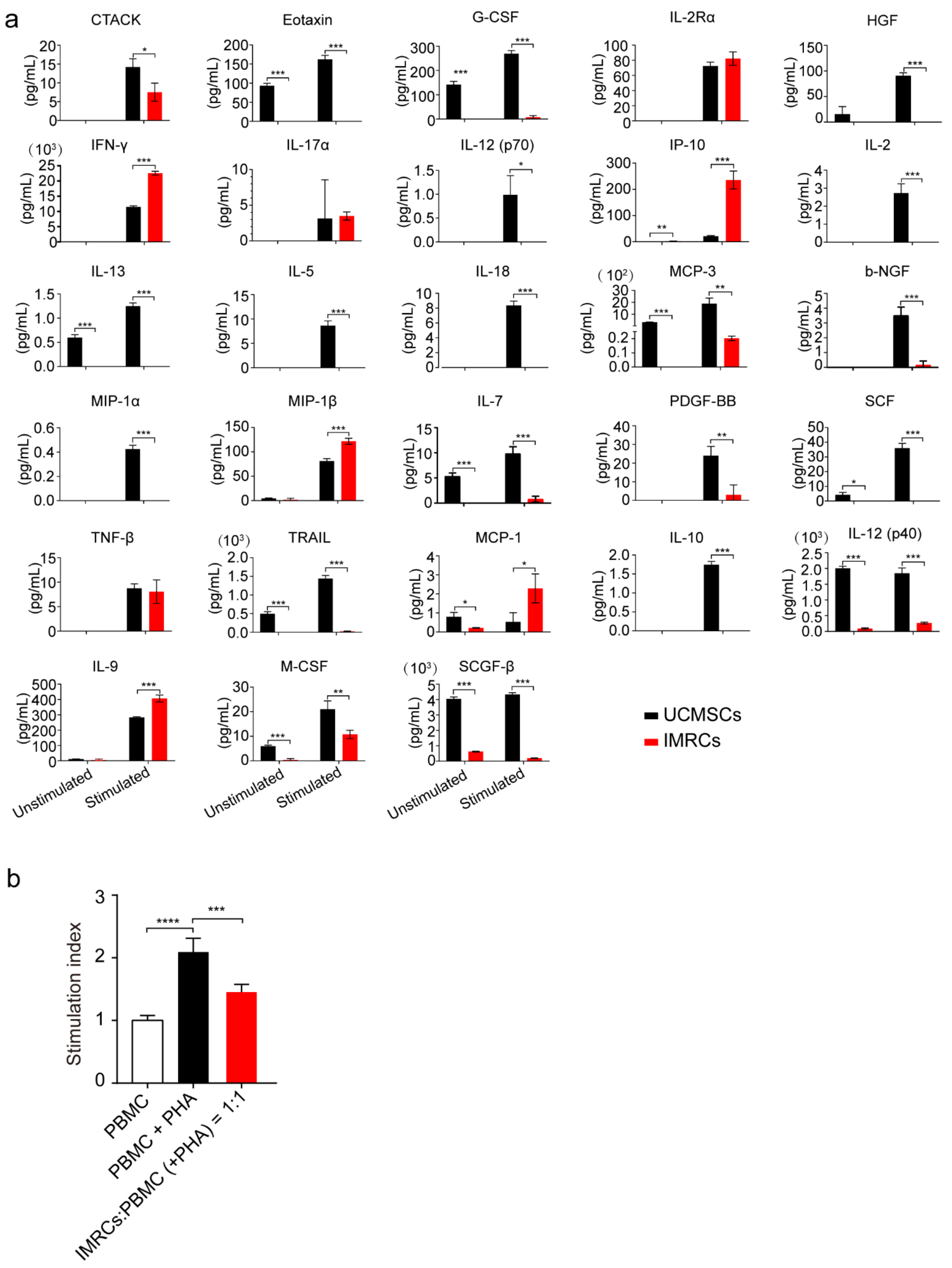

Fig. S6 IMRCs activated by IFN-γ show hyper-immunomodulatory potency.

a ELISA analysis of biologically relevant chemokines and cytokines in the secretomes of unstimulated or stimulated IMRCs and UCMSCs. bFGF, IL-15 and IL-16 were not detected. **b** The immunosuppressive effect of IMRCs on phytohaemagglutinin (PHA)-stimulated PBMCs' proliferation when cocultured together at a ratio of 1:1 (2×10^5 IMRCs vs 2×10^5 PBMCs). * $p <$

0.05, ** $p < 0.01$, *** $p < 0.001$, **** $p < 0.0001$; data are represented as the mean ± SEM.
