# Supplementary material for: Immunity-and-matrix-regulatory cells derived from human embryonic stem cells safely and effectively treat mouse lung injury and fibrosis
Source: Cell Res. 2020 Jun 16;30(9):794–809. doi: 10.1038/s41422-020-0354-1 (PMC7296193; doi:10.1038/s41422-020-0354-1)
Supplement: Supplementary file 7 — Supplementary Figure S7 [file 41422_2020_354_MOESM7_ESM.pdf]

Figure S7

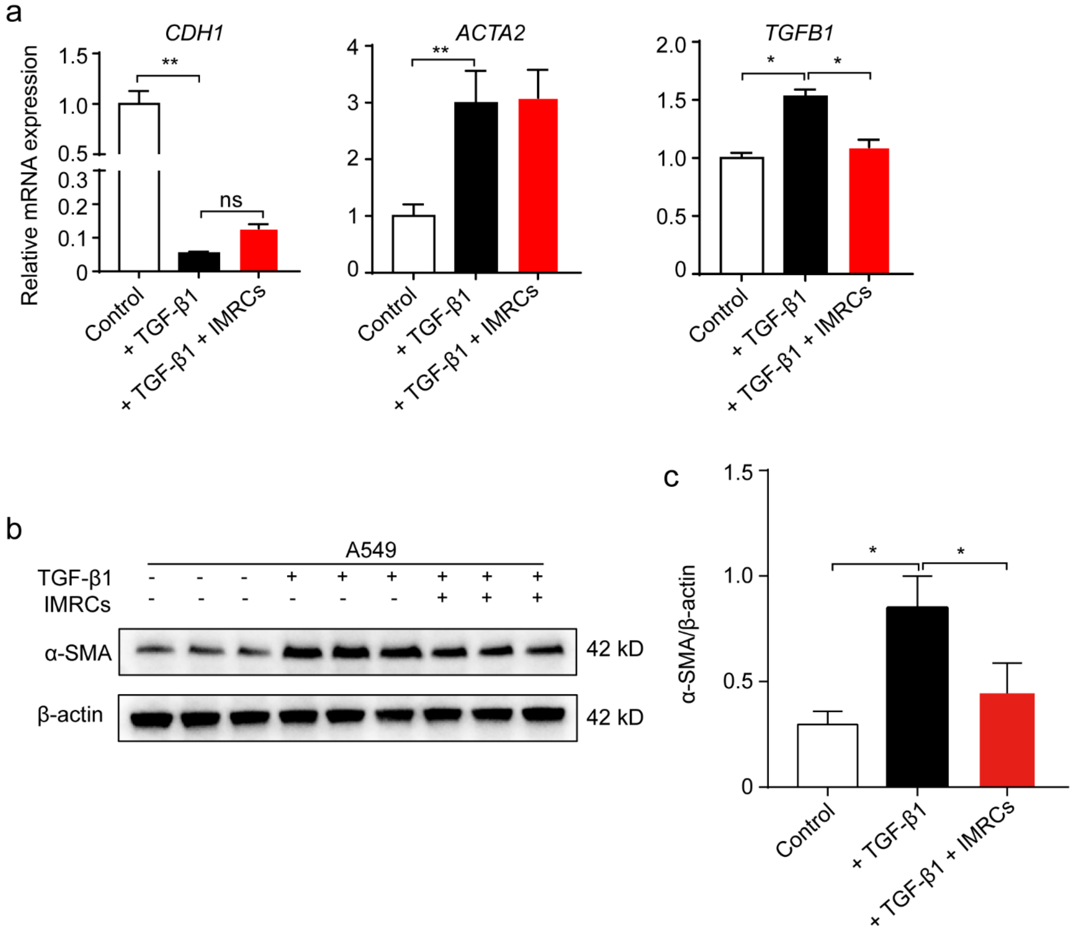

Fig. S7 IMRCs reduce the pro-fibrotic effects of TGF-β1.

a Quantitative PCR for *CDH1*, *ACAT2* and *TGFB1* mRNA in A549 cells, with or without 10 ng/mL TGF-β1 and IMRCs conditioned media treatment for 48 h. **b** Western blot for α-SMA protein expression in A549 cells, with or without 10 ng/mL TGF-β1 and IMRC conditioned media treatment for 48 h. **c** Quantification of the relative α-SMA protein expression levels in **(b)**. * $p < 0.05$, ** $p < 0.01$; data are represented as the mean ± SEM.
