# Supplementary material for: Immunity-and-matrix-regulatory cells derived from human embryonic stem cells safely and effectively treat mouse lung injury and fibrosis
Source: Cell Res. 2020 Jun 16;30(9):794–809. doi: 10.1038/s41422-020-0354-1 (PMC7296193; doi:10.1038/s41422-020-0354-1)
Supplement: Supplementary file 8 — Supplementary Figure S8 [file 41422_2020_354_MOESM8_ESM.pdf]

Figure S8

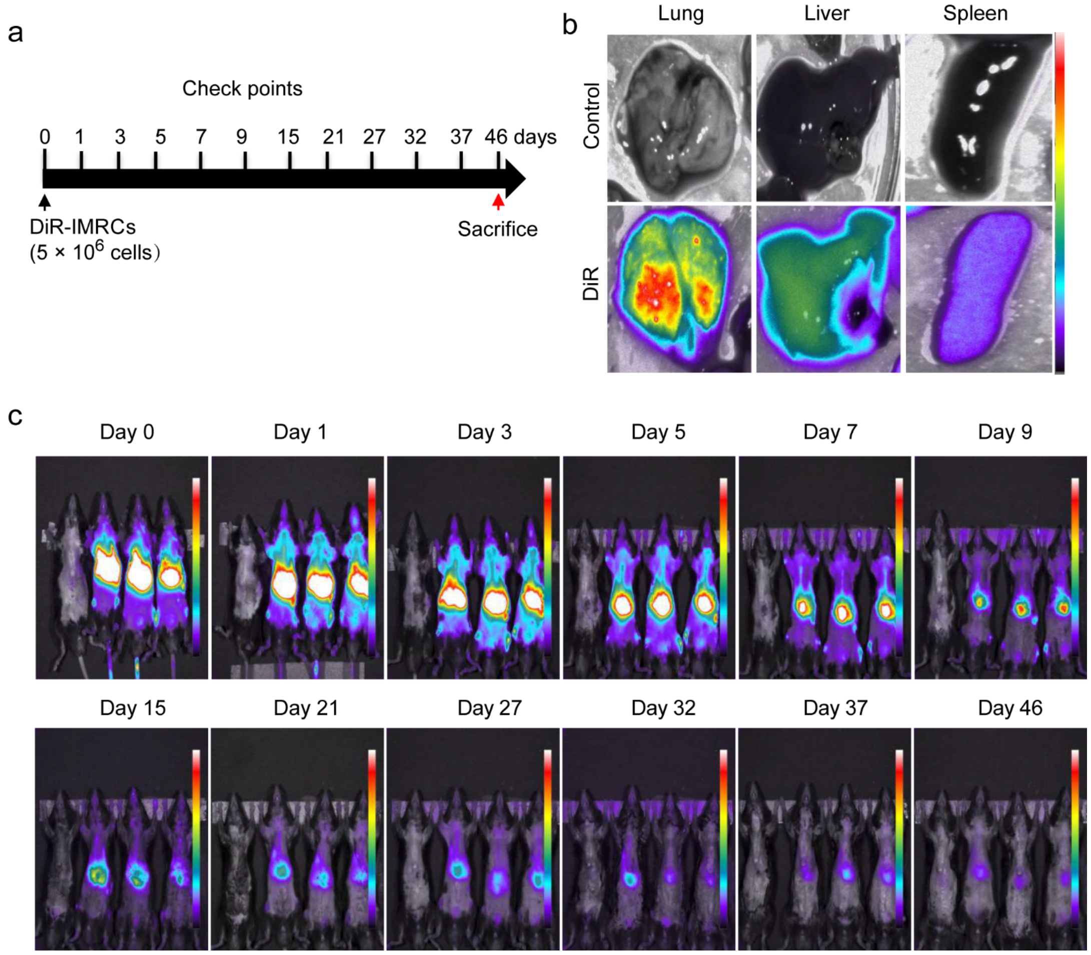

Fig. S8 Evaluation of the safety of IMRCs transfusion.

a Diagram of the animal experimental protocol. Mice were monitored using an in vivo imaging system at day 0, day 1, day 3, day 5, day 7, day 9, day 15, day 21, day 27, day 32, day 37 and day 46 after transplantation of DiR-labeled IMRCs. **b** In vivo imaging of the biodistribution of DiR far red fluorescence in the lung, liver and spleen, after injection of DiR-labeled IMRCs. **c** In vivo imaging of the biodistribution of DiR far red fluorescence after injection of DiR-labeled IMRCs.
