# Supplementary material for: Immunity-and-matrix-regulatory cells derived from human embryonic stem cells safely and effectively treat mouse lung injury and fibrosis
Source: Cell Res. 2020 Jun 16;30(9):794–809. doi: 10.1038/s41422-020-0354-1 (PMC7296193; doi:10.1038/s41422-020-0354-1)
Supplement: Supplementary file 9 — Supplementary Figure S9 [file 41422_2020_354_MOESM9_ESM.pdf]

Figure S9

a

Acute toxicity test in cynomolgus monkeys: safety analysis (after 6 months)

Group	Number of monkey	Dose (cells/kg)	Weight	Ophthalmology examination	Vital signs
High dose	1	1×10^8	Normal	Normal	Normal
Medium dose	1	0.26×10^8	Normal	Normal	Normal
Low dose	1	0.026×10^8	Normal	Normal	Normal

b

Long-term toxicity test in cynomolgus monkeys: safety analysis (one injection per week until 22 injections)

Group	Number of monkey	Dose (cells/kg)	Weight	Body temperature	Food intake	All organs' weight
Saline	Female (3) Male (3)	10 mL / monkey	Normal	Normal	Normal	Normal
Low dose	Female (3) Male (3)	0.26×10^7	Normal	Normal	Normal	Normal
High dose	Female (3) Male (3)	1×10^8	Normal	Normal	Normal	Normal

Fig. S9 Evaluation of the safety of IMRCs transfusion.

a Acute safety analysis of cynomolgus monkeys (*Macaca fascicularis*) injected with a low (0.026×10^8), medium (0.26×10^8) or high (1×10^8) dose of IMRCs after 6 months. **b** Long-term safety analysis of cynomolgus monkeys injected with a low (2.6×10^6) or high (1×10^8) dose of IMRCs, or saline, once a week for 22 times.
