# Supplementary material for: Immunity-and-matrix-regulatory cells derived from human embryonic stem cells safely and effectively treat mouse lung injury and fibrosis
Source: Cell Res. 2020 Jun 16;30(9):794–809. doi: 10.1038/s41422-020-0354-1 (PMC7296193; doi:10.1038/s41422-020-0354-1)
Supplement: Supplementary file 10 — Supplementary Figure S10 [file 41422_2020_354_MOESM10_ESM.pdf]

Figure S10

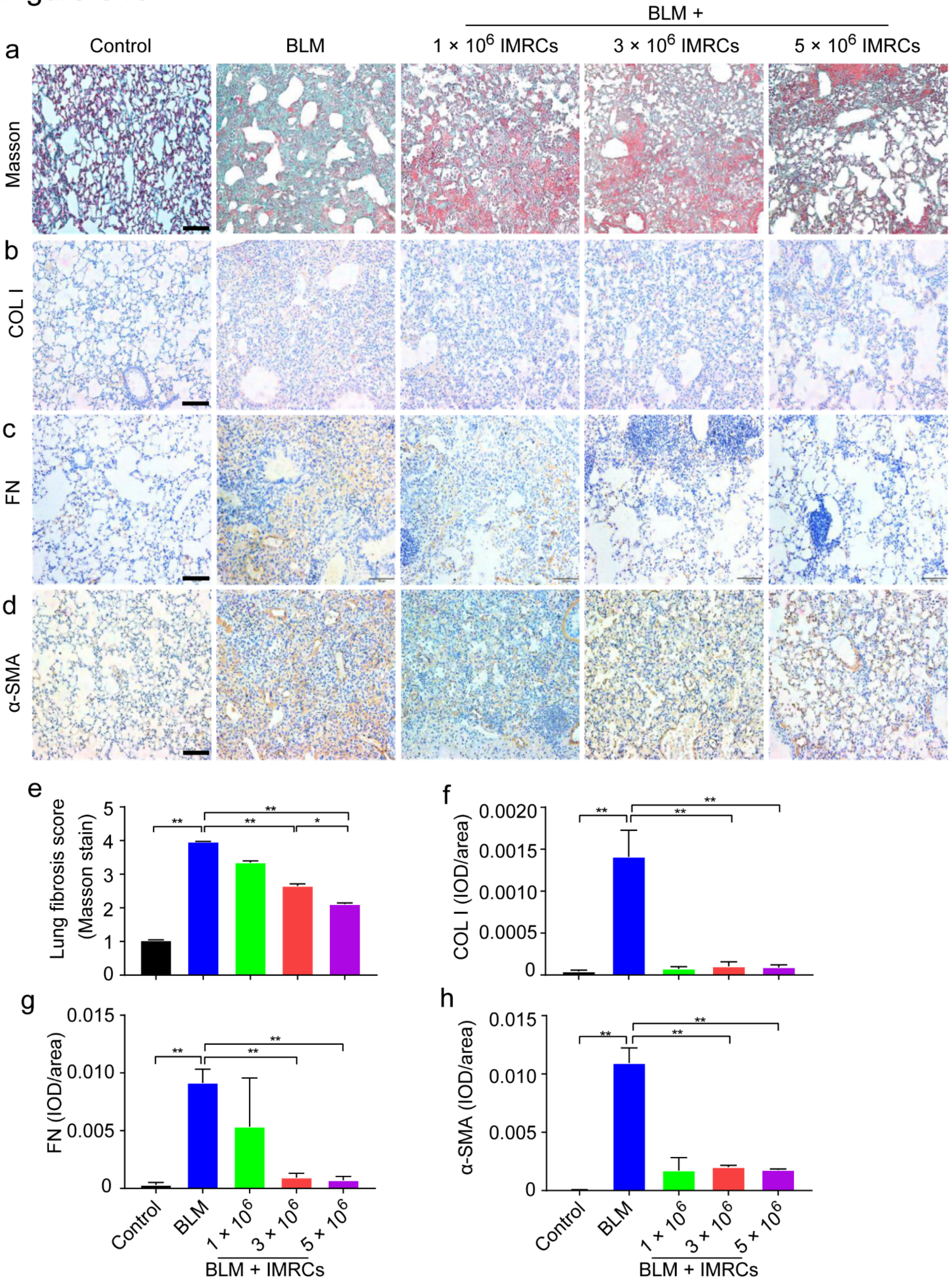

Fig. S10 IMRC transfusion treats lung injury and fibrosis dose-dependently.

a Representative lung sections of all treatment groups after Masson's trichrome staining (× 200). BLM, bleomycin. **b-d** Immunohistochemistry staining (brown) for the protein expression of **(b)** Collagen I (COL I), **(c)** Fibronectin (FN) and **(d)** α-smooth muscle actin (α-SMA), in mice receiving different interventions. **e** Lung fibrosis score, based on Masson's trichrome staining of lung sections in mice receiving different interventions. **f-h** Quantification of the immunohistochemistry staining

for **(f)** Collagen I, **(g)** Fibronectin, and **(h)** α-SMA. * $p < 0.05$, ** $p < 0.01$; data are represented as the mean ± SEM.
