# Supplementary material for: Immunity-and-matrix-regulatory cells derived from human embryonic stem cells safely and effectively treat mouse lung injury and fibrosis
Source: Cell Res. 2020 Jun 16;30(9):794–809. doi: 10.1038/s41422-020-0354-1 (PMC7296193; doi:10.1038/s41422-020-0354-1)
Supplement: Supplementary file 11 — Supplementary Figure S11 [file 41422_2020_354_MOESM11_ESM.pdf]

Figure S11

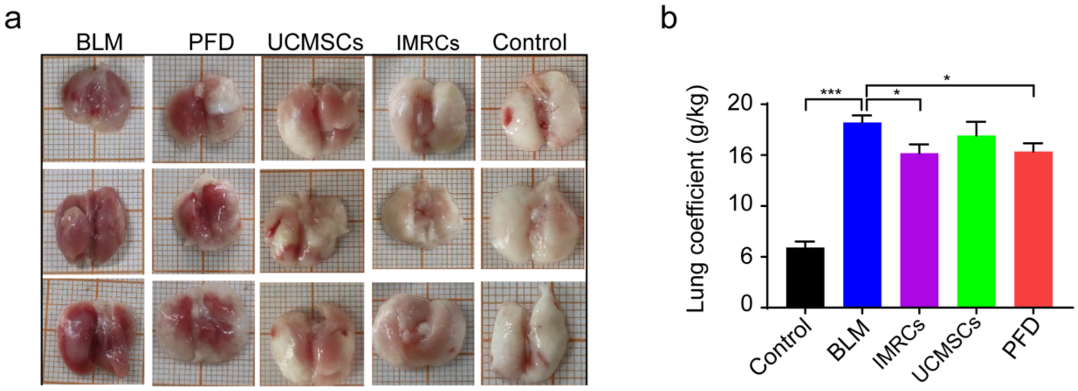

Fig. S11 IMRC treatment of lung injury and fibrosis is superior to UCMSC and pirfenidone injections.

a Representative images of whole lung from all treatment groups. One square, 1 mm. **b** Lung coefficient (wet lung weight/total body weight) of all treatment groups. * $p < 0.05$, ** $p < 0.01$, *** $p < 0.001$; data are represented as the mean ± SEM.
