# Supplementary material for: Immunity-and-matrix-regulatory cells derived from human embryonic stem cells safely and effectively treat mouse lung injury and fibrosis
Source: Cell Res. 2020 Jun 16;30(9):794–809. doi: 10.1038/s41422-020-0354-1 (PMC7296193; doi:10.1038/s41422-020-0354-1)
Supplement: Supplementary file 12 — Supplementary Figure S12 [file 41422_2020_354_MOESM12_ESM.pdf]

Figure S12

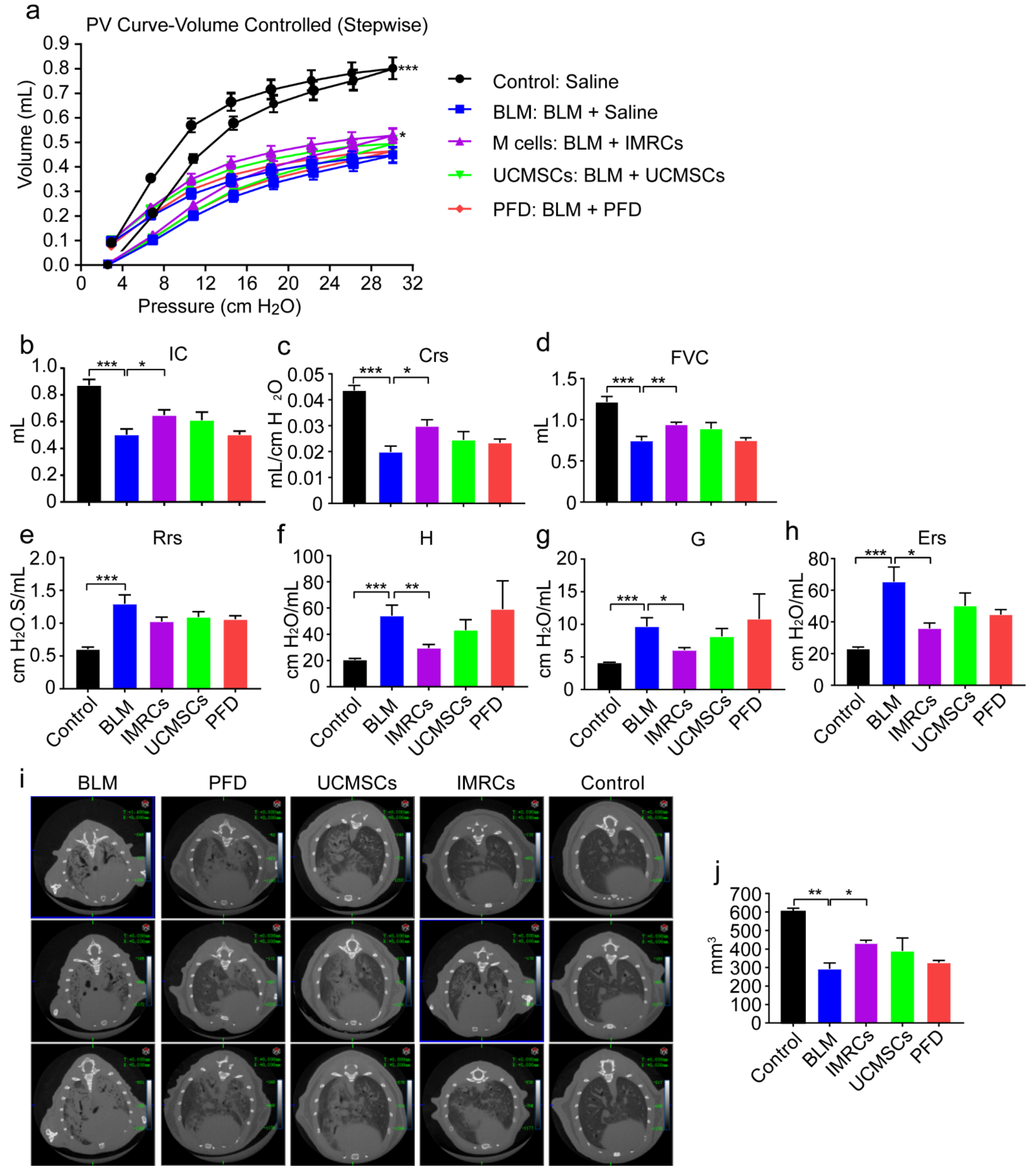

Fig. S12 IMRC treatment of lung injury and fibrosis is superior to UCMSC and pirfenidone injections.

a-h Lung mechanical function measured by FlexiVent at day 21, showing PV curves, IC, Rrs, Crs, Ers, G, H and FVC of all the experimental groups. PFD, pirfenidone; PV, pressure-volume; IC, inspiratory capacity; Rrs, respiratory resistance; Crs, static compliance; Ers, elastic resistance; G, tissue damping; H, tissue elasticity; FVC, forced vital capacity. **i-j** Micro-CT scans of the mouse lung and lung volume, in all the experimental groups. * $P < 0.05$; ** $P < 0.01$; *** $P < 0.001$.
