# Supplementary material for: Immunity-and-matrix-regulatory cells derived from human embryonic stem cells safely and effectively treat mouse lung injury and fibrosis
Source: Cell Res. 2020 Jun 16;30(9):794–809. doi: 10.1038/s41422-020-0354-1 (PMC7296193; doi:10.1038/s41422-020-0354-1)
Supplement: Supplementary file 13 — Supplementary Table S1 [file 41422_2020_354_MOESM13_ESM.pdf]

Supplementary Table S1 Biological safety analysis of the human embryonic stem cells (hESCs)-derived immunity- and matrix-regulatory cells (IMRCs)[a].

**Sterility and pathogen[b]**	**IMRCs**
**[Identification tests]**	
Cell morphology	Adherent cells in monolayer, showing fibroblast-like morphology
Isozyme analysis	B type of human origin
Short tandem repeats (STRs)	Expressing 16 STR loci, each STR locus has 1-2 alleles. STR data is consistent with its original hESCs.
**[Bacteria and fungi]**	Negative
**[Mycoplasma]**	Negative
**[Exogenous virus test - in vitro]**	
Cell observation	Cell morphology normal
Hemadsorption test	Negative
Hemagglutination test	Negative
**[Exogenous virus test - in vivo]**	
Cell inoculation in suckling mice	Survival rate > 80%
Cell inoculation in adult mice	Survival rate > 80%
Cell inoculation in guinea pigs	Survive, no tuberculosis
Cell inoculation in rabbits	Survive, no abnormality
Survival rate of 5- to 6-day-old chick embryos	Survival rate 100%
Survival rate of 9- to 11-day-old chick embryos	Survival rate 100%
Hemagglutination test of 9- to 11-day-old chick embryo allantoic fluid	Negative
**[Human virus test]**	
Human Immuno Deficiency Virus I (HIV-I)	Negative
Human Hepatitis B virus (HBV)	Negative
Human Hepatitis C virus (HCV)	Negative

Human Cytomegalo Virus (HCMV)	Negative
Epstein-Barr virus (EBV)	Negative
Human Papillomavirus (HPV)	Negative
Human herpes virus 6, 7 (HHV-6, 7)	Negative
Human Parvovirus B19	Negative
**[Bovine virus test]**	
Bovine parvovirus	Negative
Bovine adenovirus	Negative
**[Porcine virus test]**	
Porcine parvovirus	Negative
Porcine torque teno virus	Negative
**[Retrovirus test]**	
Reverse transcriptase activity	Negative
**[Immunological response test]**	
Lymphocyte proliferation inhibition rate	92.8%, coculture of IMRCs and peripheral blood monocytes (PBMCs) in the ratio of 1:5
Specific subsets of lymphocytes test	
Th1 lymphocyte proliferation inhibition rate	45.2%, coculture of IMRCs and PBMCs in the ratio of 1:5
Th17 lymphocyte proliferation inhibition rate	48.0%, coculture of IMRCs and PBMCs in the ratio of 1:5
Treg lymphocyte proliferation inhibition rate	13.8%, coculture of IMRCs and PBMCs in the ratio of 1:5
TNF-α secretion of lymphocyte inhibition rate	91.8%, coculture of IMRCs and PBMCs in the ratio of 1:5
**[Biological effectiveness test]**	
CD73 (%)	98.9
CD90 (%)	98.8
CD105 (%)	95.8

CD29 (%)	99.7
HLA-ABC (%)	99.5
CD11 (%)	< 0.1
CD19 (%)	< 0.1
CD34 (%)	< 0.1
CD45 (%)	< 0.1
HLA-DR (%)	< 0.1
**[Pluripotent cells residuals]**	
TRA-1-60$^+$ proportion assay by FACS	< 0.1%
SSEA-4$^+$ proportion assay by FACS	0.3%
TRA-1-81$^+$ proportion assay by FACS	< 0.1%
OCT4 expression assay by immunofluorescence	Negative, while its original hESCs is positive
NANOG expression assay by immunofluorescence	Negative, while its original hESCs is positive
OCT4 expression assay by PCR	Negative, while its original hESCs is positive
Nanog expression assay by PCR	Negative, while its original hESCs is positive
Teratoma formation in SCID mice	Six weeks after cell inoculation in SCID mice, no teratoma formation was observed, while its original hESCs is positive
**[Biopreparate test]**	
Endotoxin assay	≤ 0.5 EU/mL
Bovine serum albumin residuals	< 50 ng/mL

[a]This table is translated from NIFDC report (NO. SH201905849).

[b]The "Pharmacopoeia of the People's Republic of China, Edition 2015, Volume III" was used as a reference for the testing methods.
