# Supplementary material for: Immunity-and-matrix-regulatory cells derived from human embryonic stem cells safely and effectively treat mouse lung injury and fibrosis
Source: Cell Res. 2020 Jun 16;30(9):794–809. doi: 10.1038/s41422-020-0354-1 (PMC7296193; doi:10.1038/s41422-020-0354-1)
Supplement: Supplementary file 14 — Supplementary Table S2 [file 41422_2020_354_MOESM14_ESM.pdf]

Supplementary Table S2 Primer sequences used in this study.

Gene name	Forward (5’-3’)	Reverse (5’-3’)
MMP1	TGCTTCCCTGAGACCCAGTT	GATCACTTCTTTCCTTTGCATCAAG
IDO1	GCCAGCTTCGAGAAAGAGTTG	ATCCCAGAACTAGACGTGCAA
CDH1	AAAGGCCCATTTCCTAAAAACCT	TGCGTTCTCTATCCAGAGGCT
ACTA2	AAAAGACAGCTACGTGGGTGA	GCCATGTTCTATCGGGTACTTC
COLLAGEN I	GAGGGCCAAGACGAAGACATC	CAGATCACGTCATCGCACAAC
COLLAGEN II	TGGACGCCATGAAGGTTTTCT	TGGGAGCCAGATTGTCATCTC
TGFβ1	CTAATGGTGGAAACCCACAACG	TATCGCCAGGAATTGTTGCTG
FIBRONECTIN	AGGAAGCCGAGGTTTTAACTG	AGGACGCTCATAAGTGTCACC
GAPDH	CTCTGCTCCTCCTGTTCGAC	CGACCAAATCCGTTGACTCC
